# Magnetron Sputtering as a Versatile Tool for Precise Synthesis of Hybrid Iron Oxide–Graphite Nanomaterial for Electrochemical Applications

**DOI:** 10.3390/nano14030252

**Published:** 2024-01-24

**Authors:** Fee Käufer, Antje Quade, Angela Kruth, Heike Kahlert

**Affiliations:** 1 Institute of Biochemistry, University of Greifswald, 17489 Greifswald, Germany; hkahlert@uni-greifswald.de; 2 Leibniz Institute for Plasma Science and Technology, 17489 Greifswald, Germany; quade@inp-greifswald.de (A.Q.); angela.kruth@inp-greifswald.de (A.K.)

**Keywords:** iron oxides, graphite, hybrid nanomaterial, RF sputtering, reactive sputtering, PVD

## Abstract

Iron oxide nanomaterials are promising candidates for various electrochemical applications. However, under operating conditions high electric resistance is still limiting performance and lifetime. By incorporating the electronically conductive carbon into a nanohybrid, performance may be increased and degeneration due to delamination may be prevented, eliminating major drawbacks. For future applications, performance is an important key, but also cost-effective manufacturing suitable for scale-up must be developed. A possible approach that shows good potential for up-scale is magnetron sputtering. In this study, a systematic investigation of iron oxides produced by RF magnetron sputtering was carried out, with a focus on establishing correlations between process parameters and resulting structural properties. It was observed that increasing the process pressure was favourable with regard to porosity. Over the entire pressure range investigated, the product consisted of low-crystalline Fe_3_O_4_, as well as Fe_2_O_3_ as a minor phase. During sputtering, a high degree of graphitisation of carbon was achieved, allowing for sufficient electronic conductivity. By means of a new alternating magnetron sputtering process, highly homogeneous salt-and-pepper-type arrangements of both nanodomains, iron oxide and carbon were achieved. This nano-containment of the redox-active species in a highly conductive carbon domain improves the material’s overall conductivity, while simultaneously increasing the electrochemical stability by 44%, as confirmed by cyclic voltammetry.

## 1. Introduction

Iron oxide nanomaterials are of growing interest due to their properties and broad range of potential applications, especially in electrochemistry. They exhibit high reactivity, good corrosion resistance and environmental friendliness, as well as being abundant in nature and cost efficient [1,2,3]. At the same time, their comparably low electronic conductivity, volume change and resulting instability during electrochemical cycling are challenging for electrochemical applications. One way to address these drawbacks and improve the performance of iron oxides is to incorporate conductive carbon materials, resulting in the formation of nanohybrid materials. This way, favourable properties of iron oxides are combined with the high electrical conductivity of carbon materials [4,5,6], enabling better suitability for electrochemical applications.

One increasingly important application of iron oxide-based nanomaterials is water purification via the Fenton reaction. In combination with H_2_O_2_, iron oxides act as a catalyst to produce reactive hydroxyl radicals. These can degrade any electron-rich organic molecule to H_2_O, CO_2_ and inorganic ions [7,8]. For a heterogeneous electro Fenton approach the redox-active material may be immobilised and coated onto a cathode support. Immobilisation eliminates many drawbacks of homogeneous electro-Fenton systems, where iron is introduced as a soluble salt since no iron is lost from the redox-active system and no iron sludge is produced. Furthermore, a neutral working pH is possible because the observed formation of iron oxide hydroxides at higher pH is not occurring anymore as it is stabilized at the electrode surface [9,10]. Fe_2_O_3_ may also be applied as anode material in lithium-iron batteries and shows a high specific capacity of 1007 mAh g^−1^. It is also a suitable anode material for potassium iron batteries [1,2,5].

There are various methods for the preparation of iron oxide nanomaterials, whereby co-precipitation in alkaline solution is the most common technique [11,12,13,14]. In this process, iron salts are dissolved in an aqueous solution, and iron oxides precipitate as pH increases [11]. A study in which graphene oxides have been incorporated into the iron oxide material during co-precipitation has also been reported. For this purpose, an aqueous graphene suspension was mixed with the iron salt solution and co-precipitation occurred during the increase in pH. To extract the material, the mixture must be heated, filtered and dried [3]. Hence, high process complexity, time, as well as costs are given. Also, the product does not contain hybridized particles but a plain physical mixture with poor interfaces between nanoparticles.

Another method for producing iron oxide nanomaterials is magnetron sputtering, a common physical vapor deposition method. It is well suited for the creation of nanohybrids since stable interfaces are formed in situ and the composition, elemental distribution and nanostructure may be controlled by process parameters. This method is also of high suitability for roll-to-roll processes in industrial manufacturing and does not use any harmful chemicals, which is an advantage over wet chemical approaches [15,16,17]. During magnetron sputtering, by bombarding a target with high-energy argon ions, atoms are ejected from the target and deposited onto a substrate, giving rise to a highly versatile tool and cost-effective for thin-film preparation [15,16]. By adjusting process parameters, precise control of the material’s properties with regard to suitability for electrochemical applications is achieved [18].

Iron oxide films can be derived by sputtering from an iron oxide target or by reactive sputtering in oxygen from an elemental iron target [19,20,21,22]. However, there are no reports on sputtering processes for the synthesis of iron oxide–graphite nanohybrids for electrochemical applications so far. First studies to overcome the challenges of wet-chemical synthesis were reported on ion beam sputtering. In a study, iron and carbon were deposited successfully; however, extensive post-reaction treatments were required to achieve hybridization and prevent de-mixing [23].

In this study, a novel combined magnetron sputtering process for the production of iron oxide confined in a nanographite containment is developed. This approach is expected to allow for an increase in conductivity, as well as address instability due to volume change during redox reactions. Since process pressure is one of the most important parameters that affect the properties of sputtered materials, emphasis is given to the observation of correlations between pressure and the microstructure of the nanomaterial.

## 2. Materials and Methods

### 2.1. Materials Fabrication

The nanomaterial was deposited by a radio frequency (RF) magnetron sputtering method, onto a graphitised carbon felt (CF) with a microporous layer (H24C5, Freudenberg, Weinheim, Germany) that was placed on a rotating table. The reactor chamber was equipped with a 2″ diameter iron target (99.95%, EVOCHEM Advanced Materials GmbH, Offenbach, Germany) and a 2″ diameter carbon target (99.9%, MaTeck GmbH, Jülich, Germany). The sputtering process was carried out at room temperature once a pressure of 10^−4^ bar was reached.

The iron target was conditioned by sputtering in an argon atmosphere at 200 W at a closed shutter over a period of 60 s in order to remove surface contaminants. For reactive sputtering of iron, the chamber was purged with a process gas mixture of argon (99.999%) and oxygen (99.999%) at a ratio of 5 Ar: 8 O_2_. Iron oxide was deposited for six hours onto the carbon felt at 100 W at varying pressures of 5 Pa, 6.5 Pa and 8 Pa, giving rise to a layer of approx. 40 nm in layer thickness independently from pressure.

For the deposition of iron oxide–carbon nanohybrids an alternative magnetron sputtering process was applied and an additional step for carbon deposition was introduced to the process. Prior to this process step, evacuation of the chamber occurred and pure argon was introduced as the process gas at a flow of 50 sccm. The carbon target was conditioned at 200 W and at a closed shutter over a period of 60 s to remove surface contaminants. The deposition process was carried out over 20 min, at 1 Pa and 50 W, giving rise to layer thicknesses of 37 nm of carbon. This equals a deposition rate of 112 nm per h. The carbon deposition step was alternated with the deposition of 20 nm of iron oxides, carried out at a process pressure of 6.5 Pa. This combined procedure was repeated three times to obtain a hybrid material consisting of carbon and iron oxide nanodomains.

### 2.2. Structural, Morphological and Chemical Characterisation

Characteristics of the nanomaterial with regard to stoichiometry and structure were determined using Inductively Coupled Plasma with Optical Emission Spectrometry (ICP-OES), X-ray Photoelectron Spectroscopy (XPS), X-ray diffraction (XRD), Raman Spectroscopy, Scanning Electron Microscopy (SEM) and energy dispersive X-ray spectroscopy (EDX). All structural characterisations were carried out on samples with 6 h deposition time of iron oxide.

Layer thicknesses were measured on samples deposited onto soda–lime microscope slides (Duran Group, Wertheim, Germany). Therefore, a VK-X3000 Keyence Laser Microscope (Keyence, Osaka, Japan) was used for white light interferometry to measure the average step height between an uncoated reference plane and the layer.

For determination of the specific iron loading per area of carbon cloths, four pieces 5 mm in diameter were cut from each coated sample. These were dried at 40 °C before being digested. Therefore, each one was immersed in a mixture of 4.5 mL of concentrated HNO_3_ and 1.5 mL of concentrated HCl and stirred for two hours. After 30 min in an ultrasonic bath, the solution was filled up to 25 mL and then filtered via 0.45 µm syringe filters. Samples were tested for iron with ICP-OES (Optima 2100 DV, PerkinElmer, Nowwalk, CT, USA). For calibration, solutions in a range between 0.06 mgL^−1^ and 0.14 mgL^−1^ of iron(III) ions were prepared from a standard solution (1000 µgL^−1^ (Fe(NO_3_)_3_)∙9H_2_O in 5% HNO_3_, Specpure, AlfaAesar, Ward Hill, MA, USA). Triple measurements were performed for each sample at a wavelength of 238.204 nm. For evaluation and presentation of the results, the program WinLab32 (PerkinElmer, Nowwalk, CT, USA) was used.

XPS measurements were performed using an Axis Supra DLD electron spectrometer (Kratos Analytical, Manchester, UK). Analysis was conducted with a monochromatic Al Kα X-ray source (1486.6 eV), at 150 W on an area with a diameter of approximately 250 µm. Three different spots of the sample were measured. For data processing, peak adjustment with a Gaussian–Lorentzian shape and Shirley background subtraction CasaXPS software, version 2.14dev29 (Casa Software Ltd., Teignmouth, UK) was used.

A StadiP device (STOE, Darmstadt, Germany) with Mo Kα_1_ radiation and a Mythen2 2K detector was used for XRD. The sputtered iron oxide materials were mechanically removed from the substrate, ground using an agate mortar and pestle and deposited onto acetate foil for transmission studies. Diffraction patterns were recorded over a range of *2θ* angles from 5° to 60°, at a step width of 0.02° and a collection time of 1200 s per step. The evaluation was performed using STOE WinXPOW software version 3.21 and the ICDD PDF-2 database.

Raman data were recorded using a confocal Renishaw in Via microscope with an Nd-YAG laser whose excitation wavelength was at 532 nm. To analyse the nanomaterial, 10 accumulations were made for 30 s each with a laser power of 1% with a 100× objective lens. A spectral range of 900–3500 cm^−1^ was investigated for carbon analysis and one of 200–1500 cm^−1^ for iron oxides. Data were evaluated using WiRE 3.4 software (Renishaw, Wotton-under-Edge, UK).

The morphology was investigated by a JEOL JSM-7500F SEM (JOEL Ltd., Akishima, Japan) with an in-lense secondary electron detector. Images were taken applying an acceleration voltage of 5 kV and an emission current of 10 µA.

For analysis of the hybrid characteristic, the sample was embedded in epoxy. The cross sections were prepared by Focussed Ion Beam using a ThermoFisher Scientific Scios2 HiVac (ThermoFisher Scientific, Waltham, MA, USA). A 2 µm protective platinum layer was deposited onto the samples by sputtering, followed by rough milling which was performed with 30 kV and 65 nA Ga ion beam, preparing a cut-out with a width of 100 µm and a depth of 30 µm. Fine polishing was continued at 3 nA. The images were taken at 10 kV and 0.2 nA with a ThermoFisher Scientific Apreo 2 SEM using the backscattering electron detector. For EDX mapping, a ThermoFisher UltraDry Premium EDS detector with 60 mm^2^ active detection area and 129 eV was applied for 60 s.

### 2.3. Electrochemical Studies

To investigate the performance of the iron oxide catalyst with carbon, resistance, electrochemical stability and activity of the material were determined. Ohmic resistance was measured using a four-point measurement device (Series 2400, Keithley Instruments, Solon, OH, USA) with five repetitions per sample.

A conventional three-electrode arrangement was used to investigate the stability of the nanomaterial under applied potential. Coated carbon felt was used as the working electrode, a Pt wire as the auxiliary electrode, and Metrohm Ag/AgCl (saturated KCl, E = 0.208 V vs. SHE) as a reference electrode. At room temperature, a 50 mM Na_2_SO_4_ solution (≥99%, Honeywe, Charlotte, NC, USA) was pumped through the felt as an electrolyte at a constant potential of −0.7 V in a flow-through system. It was applied using a µAutolab Type II (Metrohm, Herisau, Switzerland) and controlled via via Nova software version 2.1.1 (Metrohm, Herisau, Switzerland). In order to study degradation, samples were collected at the outlet of the flow cell for analysis by ICP-OES and compared with standards at according concentrations.

The electrochemical activity during redox cycling was determined by recording cyclic voltammograms (CV). Therefore, a Pt sheet was used as an auxiliary electrode and the same reference electrode as before. Experiments were carried out in 50 mM Na_2_SO_4_ solution in a one-pot system without flow. The solution was purged with nitrogen for 5 min before measurements. The same potentiostat was used to apply the potential which was scanned between −0.7 V and 0.7 V with a scan rate of 0.1 V per s, for five scans.

## 3. Results and Discussion

### 3.1. Iron Oxide Nanomaterials

For investigation of correlations between process pressure and product properties, three different pressures of 5 Pa, 6.5 Pa and 8 Pa were applied during sputtering of iron oxide. The iron loading was determined by the digestion of a specific sample area and subsequent measurement for iron via ICP-OES. Observed loadings range from 25–34 (±9) mg per cm^2^ for process periods of 6 h, showing only little variation with pressure. The homogeneity was calculated according to Danzer et al. (2001) [24]. Correspondingly, a small deviation of 0.3 mg per cm^2^ is due to the analytical procedure. A variation of 8.7 mg per cm^2^ across the electrode areas was observed due to the non-uniformity of film thickness. A variation in layer thickness is commonly observed for sputtering processes employing circular targets of limited sizes, where the coating thickness depends on the flow geometry [25]. A variation of 25% was, however, considered to be acceptable for the investigation.

**Figure 1:**
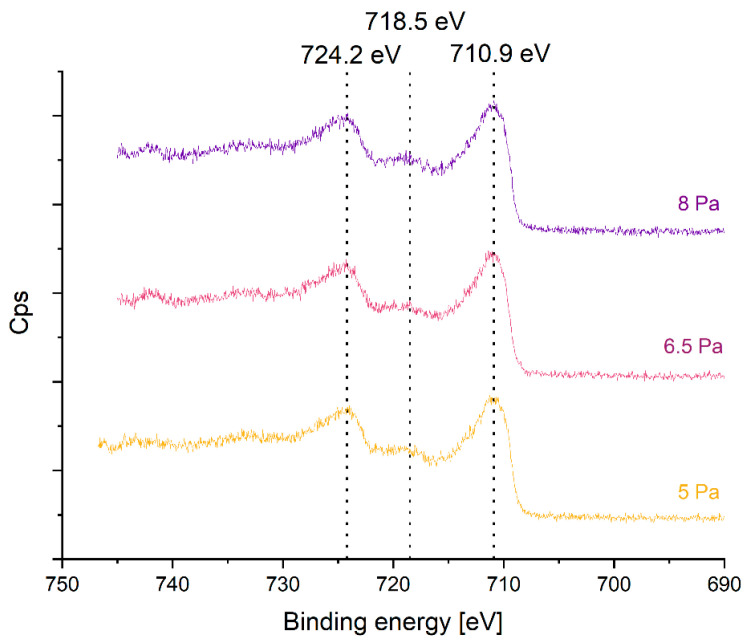
XPS spectra of iron oxides prepared at different pressures showing the Fe 2p peak.

According to XPS data, the oxidation state and bond characteristics of the deposited iron oxide’s surface are similar at all pressures (Figure 1). The Fe 2p_3/2_ peak is found at a binding energy of 710.9 eV and a satellite peak occurs at 718.5 eV (Figure S1). Pure Fe^2+^ would be expected to exhibit a binding energy of 715.5 eV and pure Fe^3+^ at 719 eV [26]. The observed value of 718.5 eV, as well as the distinct shape of the peak, indicates the presence of Fe^3+^ as a main phase. In addition, the satellite peak appears more correlated to higher binding energies [26]. The Auger parameter lies, however, between 1412 eV and 1415 eV, and hence, in a range of magnetite (Fe_3_O_4_). This leads to the assumption that the surface layer may consist of a mixture of Fe_2_O_3_ and Fe_3_O_4_ (Figure S2). However, it is important to mention that XPS only studies the surface. For used XPS instrument and x-ray source the first 7 nm are investigated.

**Figure 2:**
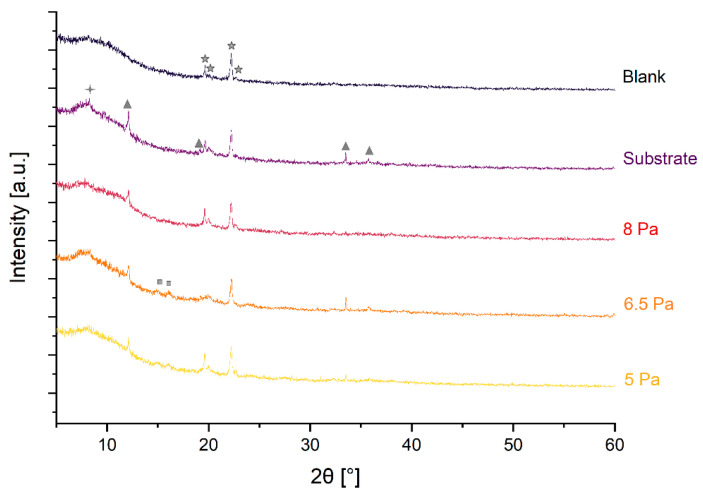
XRD patterns for iron oxide material deposited at different pressures in comparison to the uncoated substrate and a blank measurement for an empty sample holder. Peaks arise from the instrument (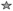), Teflon (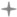), graphite (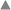) and hematite (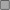).

The XRD patterns show a number of reflexes arising from the sputtered coatings as well as from a blank sample holder and the substrate (Figure 2). The presence of Teflon (PDF 00-060-1504) and graphite (PDF 00-056-0159) in the substrate is apparent. There are broad reflexes visible at 14.8° and 16.1° (Figure 2). Although these reflexes are of low intensity and broad, they may indicate the presence of hematite, α-Fe_2_O_3_, of low crystallinity [27]. The signal intensities appear to vary slightly between samples produced at different pressures; however, the intensity variation is likely to occur from a variation of sample amounts employed for the XRD study.

**Figure 3:**
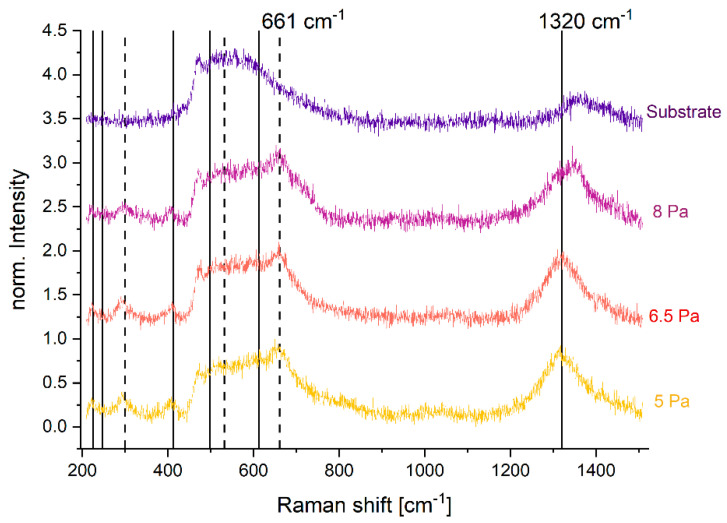
Raman spectra of iron oxides deposited on CF at different pressures with peaks indicating the presence of Fe_3_O_4_ and α-Fe_2_O_3_. Reference peak positions from literature values for α-Fe_2_O_3_ are shown by means of full line markers, with main peak position at 1320 cm^−1^. The reference peak positions for Fe_3_O_4_ are indicated by dashed line markers, with the main peak at 661 cm^−1^.

Complementary Raman data show also the presence of hematite, α-Fe_2_O_3_, with two-magnon scattering giving rise to a peak at 1320 cm^−1^. Additionally, magnetite, Fe_3_O_4_, is detected using a characteristic peak for the A_1g_ species appearing at 661 cm^−1^ (Figure 3) [28,29]. Only one peak shift for the 8 Pa sample is observed from 1320 cm^−1^ to 1345 cm^−1^ (Figure 3). As discussed later on, a stronger signal from substrate material may be given due to the higher porosity of the coating at higher pressures. Because the scattering power is much higher for hematite than for magnetite, high peak intensities are given even for smaller phase ratios of hematite [28]. From XPS, XRD and Raman analysis, it may be concluded that reactive magnetron sputtering produces a mixture of low-crystalline magnetite as a main phase as well as hematite as a minor phase.

The formation of magnetite and hematite phases by magnetron sputtering has been reported in previous studies [19,22,30]. Aubry et al. [22] showed that the amount of oxygen in the process gas influences the quantitative ratio of these two phases significantly. At small oxygen content, pure hematite is produced, and at higher oxygen content, magnetite is formed as a second phase, whereby its ratio increases with oxygen content. At higher oxygen contents, also the crystallinity of hematite and magnetite is increased [22]. Correlating to the study of Aubry et al., a comparable high ratio of process gas oxygen was given in the presented study, favouring the formation of mainly magnetite. A study by Miller et al. [19] also investigated the effect the oxygen partial pressure has during reactive sputtering of iron oxides, also taking into account substrate temperature. A correlation between those parameters and particle size and crystallinity was found. At low substrate temperature and high partial oxygen pressure, hematites of lower crystallinity and smaller particle sizes appear [19]. Results from the presented study are also in correlation with Miller et al. showing low crystallinity of magnetite and hematite phases formed at high process pressure of 5 to 8 bar.

**Figure 4:**
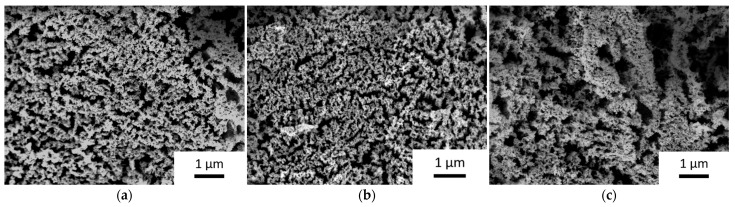
SEM pictures of iron oxide deposited by RF magnetron sputtering (**a**) at 5 Pa; (**b**) at 6.5 Pa; (**c**) at 8 Pa. All pictures were recorded at 5 kV at a working distance of 6.7 mm with a magnification of ×15,000.

From SEM images of the coated carbon felts, porous structures were observed for iron oxide materials produced at all three pressures. The particle sizes appear to be independent of the pressure, showing similar values of 66 (±7) nm at 5 Pa, 49 (±6) nm at 6.5 Pa and 56 (±5) nm at 8 Pa. However, the observed pore sizes change significantly with pressure. While pores sizes are well distributed and around 100 nm at 5 Pa, particles appear as agglomerated domains forming larger pores at a process pressure of 6.5 Pa with pore sizes reaching up to 1 µm at 8 Pa (Figure 4). This observation is consistent with other studies that reported higher porosity at increasing pressure during magnetron sputtering [31] and confirms the possibility of controlling porosity and microstructure of iron oxides by pressure during sputtering.

**Figure 5:**
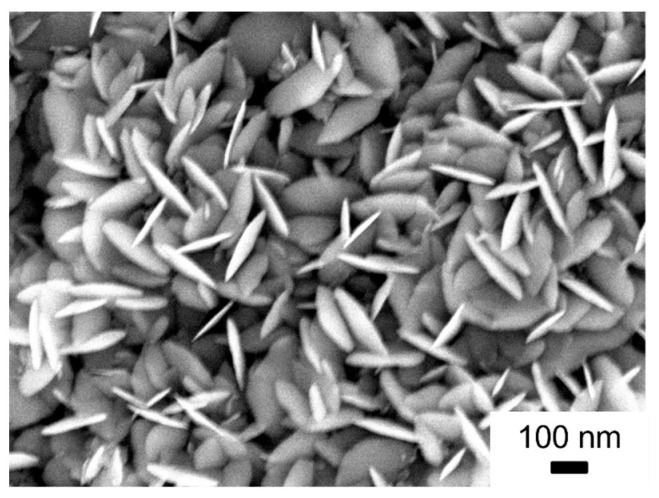
SEM pictures of iron oxide material deposited for 18 h by RF magnetron sputtering, recorded at 5 kV at a working distance of 7.8 mm with a magnification of ×65,000.

To study growth behaviour and to investigate the development of the material’s nanostructure with process time, deposition times were increased to 18 h. The SEM image of such a thick layer of sputtered iron oxide shows that particles grow in a disc-shaped manner (Figure 5). The discs are of even sizes with 194 (±14) nm in length and 22 (±3) nm in width.

The observed formation of disc-shaped particles at long sputtering times indicates that deposition occurs as island growth. This growth is also termed Volmer–Weber growth and occurs when the adhesion between deposited particles is stronger than that between particles and substrate. This way, separate islands grow, and the substrate is not uniformly covered layer by layer. As more material is added, the number of islands and their size increase until the individual features merge [32,33].

Synthesis and its parameters have been shown to influence the shape of iron oxide nanoparticles. Many different shapes have been reported [2,34,35]. A disc shape, like that produced in this study, has previously been achieved by various hydrothermal methods [36]. In comparison, sputtering avoids the production of chemical wastes with the same result as particles.

### 3.2. Carbon Nanomaterial

**Figure 6:**
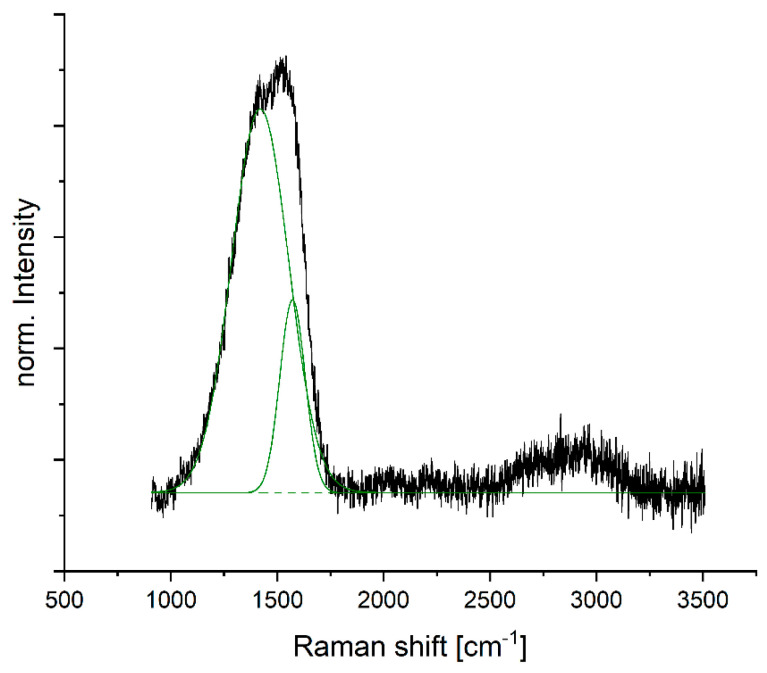
Raman spectrum (black) of deposited carbon material with indicated D and G peaks underlying (green).

Raman data collected for a magnetron sputtered carbon layer of 670 nm show the D and G peaks typical for carbon, as dominating features (Figure 6). The D peak usually appears around 1330 cm^−1^ and is known as the defect band. It is proportional to the amount of defects present in the carbon material because the band is caused by breathing vibrations of disordered graphite [37,38]. As the measured spectrum shows a large D peak, the carbon material has many defects. The broad character indicates the presence of amorphous carbon. However, it is likely not a pure phase but can also include graphite oxide [39].

The second peak is the G peak, which typically appears around 1580 cm^−1^ and is caused by sp2 site vibrations, in both chains and ring structures [38]. The presence of sp2 hybridized carbon is supported by XPS measurements, which show that more than 70% of the carbon present at the surface, is sp2 hybridized.

Further information about the material is provided by the ratio between the peaks I(D)/I(G), which is 4.5 for the carbon studied. A higher ratio indicates the presence of more aromatic rings instead of sp2 or sp3 chain bonds [38]. Therefore, it can be derived that the carbon material forming during sputtering is a carbon phase that contains many defects and aromatic rings. In terms of applications, defects can be beneficial as they increase the material’s mechanical strength as well as electrical conductivity and functionality for the formation of bonds [40]. Such carbon material is expected to be suitable as a component in nanohybrids for electrochemical applications.

**Figure 7:**
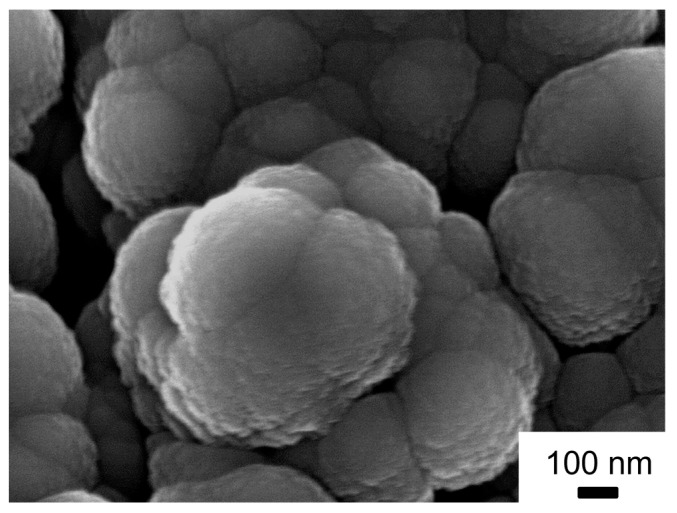
SEM picture of carbon material deposited for 6 h via RF magnetron sputtering, recorded at 5 kV at a working distance of 7.8 mm with a magnification of ×65,000.

The SEM image of such a thick layer of sputtered carbon reveals that particle growth occurs in cauliflower structures of agglomerated spherical particles of 337 (±30) nm (Figure 7). They grow to form columns of about 1 µm in diameter. This is due to Volmer–Weber growth typically observed for sputtering processes [32].

### 3.3. Hybrid Nanomaterial

**Figure 8:**
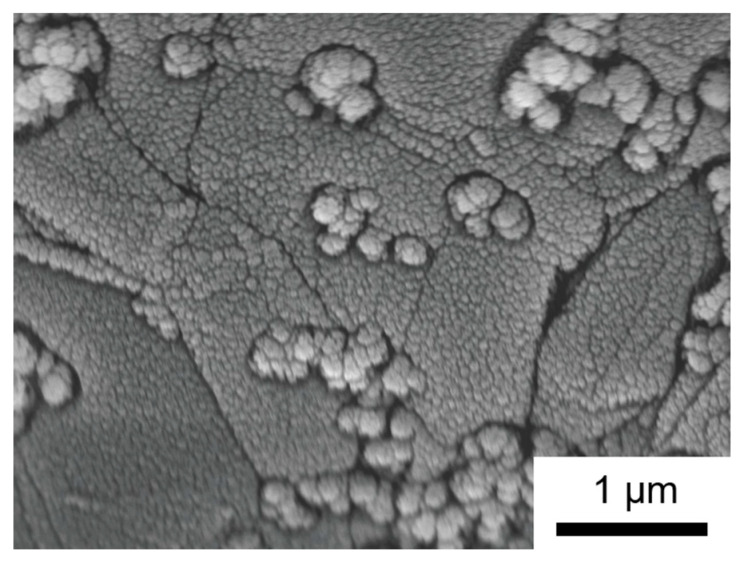
SEM picture of iron oxide–graphite hybrid material produced via alternated magnetron sputtering recorded at 5 kV with a magnification of ×25,000.

**Figure 9:**
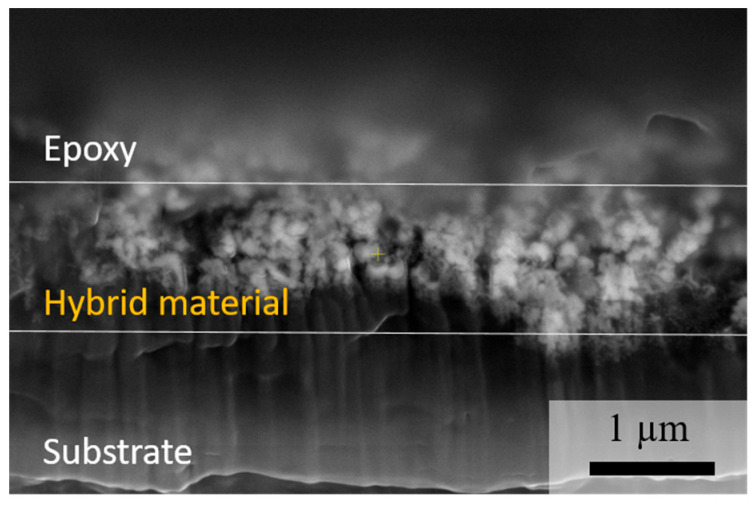
SEM picture of the cross-section of iron oxide–graphite hybrid material produced by alternated magnetron sputtering recorded at 10 kV with a magnification of ×35,000.

When iron oxides and graphite are co-deposited by alternate magnetron sputtering, the morphology of the material changes significantly in comparison to the single-phase forms. From the top view of the hybrid material, the formation of islands consisting of merged particles of sizes 54 (±2) nm is observed (Figure 8). Cross-section images verify the formation of merged particles arranged into nanocolumns, forming a porous coating (Figure 9).

**Figure 10:**
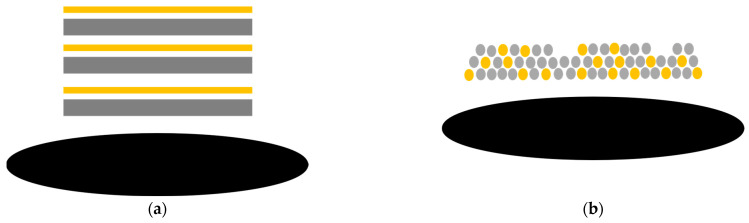
Schematic representation of iron oxide (yellow) and graphite (grey) hybrid material deposited by alternated magnetron sputtering (**a**) deposition followed by layer growth; (**b**) salt-and-pepper structure due to island growth.

Due to Volmer–Weber growth, which is observed for both processes—the deposition of iron oxides and the deposition of carbon—the carbon particles are included in the iron oxide particle formations. This means that the hybridization of iron oxide and carbon occurs as a mixture of their particles. Thus, the components of the hybrid material are evenly distributed (Figure 10). This is confirmed by EDX mapping which shows a homogeneous distribution of iron and carbon within the cross-sectioned layer (Figure S3). The more mixed character achieved by magnetron sputtering is likely to increase the effect of carbon even further.

### 3.4. Results of Electrochemical Studies

The aim of this study was the fabrication of a nanohybrid consisting of iron oxide and carbon by magnetron sputtering and assessment of properties with regard to electrochemical applications. To determine the conductivity, four-point resistance measurements were carried out on carbon felt substrates with an iron oxide coating and with the hybrid material coating, both containing the same amount of iron. Results show that the incorporation of carbon decreases the resistance from 3.8 ± 0.3 Ω for the iron oxide-coated substrates to 3.2 ± 0.2 Ω for substrates coated with the iron oxide–graphite nanohybrid. This difference is significant (*t*-test, *t* = −4.23, df = 8, *p*-value = 0.00287).

**Figure 11:**
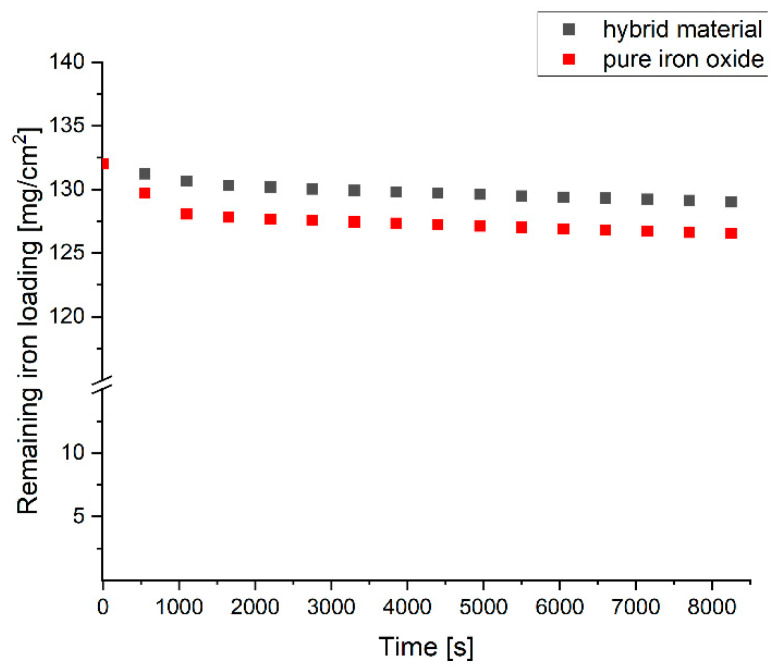
Degradation of electrode coating under operating conditions during polarisation at −0.7 V by investigation of iron loss from coating into eluent over time—comparison of pure iron oxide coating and hybrid material coating.

In order to assess stability under applied potential coated carbon felt substrates with an area of A = 6.3 cm^2^ were applied under operating conditions at −0.7 V in a three-electrode set-up. For both felts, the initial loading is 132 mg per cm^2^. The pure iron oxide coating appears to exhibit instability under these conditions, as 5.4 mg per cm^2^ of iron is eluted, which corresponds to a loss of 4.1% of the initial loading. The bulk of loss of iron occurs within the first 1000 s of process time indicating that loosely attached iron is the reason (Figure 11). In comparison, the hybrid coating also shows such an initial loss, but to a smaller extent, of 3.0 mg per cm^2^ of iron, which is equivalent to 2.3% of the initial loading. The incorporation of carbon greatly increases the initial stability of iron oxide under applied potential. This is possibly due to the nanoconfinement of the iron oxide particles by the stable carbon containment and improved fixation of the redox-active species. After 1000 s of polarisation only a minimal and gradual decay is observed. Most likely equilibrium processes during cycling are the reason. The rate is similar for both coatings.

**Figure 12:**
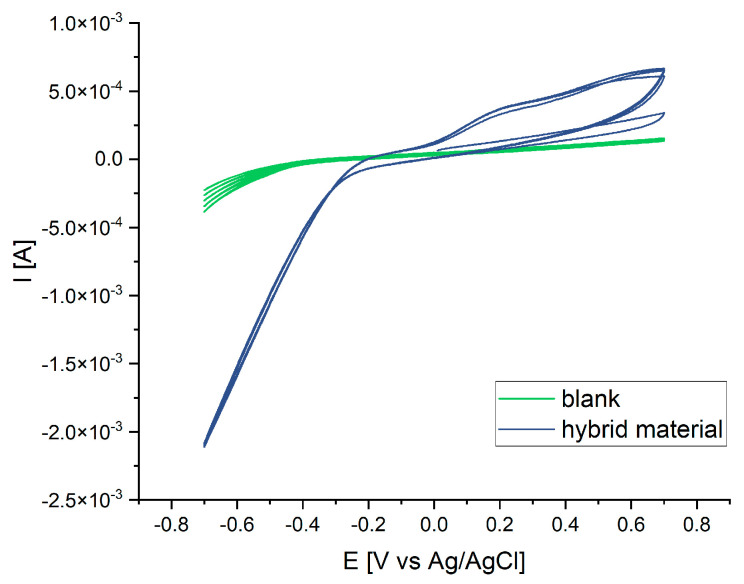
CV measurements of uncoated carbon felt (green) and carbon felt coated with iron oxide–graphite hybrid material (blue).

Cyclic voltammetry was carried out for the assessment of redox properties and stability of the material. From the CV of an uncoated substrate, no peaks are apparent, whilst an oxidation peak can be observed at 0.21 V vs. Ag/AgCl for the felt coated with the iron oxide–graphite hybrid material (Figure 12). It does not occur at the first scan that starts at 0 V but at each subsequent scan. Under reductive conditions (i.e., decreasing potentials), a steep current drop occurs instead of a peak (Figure 12). The oxidation peak present in the CV is caused by the oxidation of Fe^2+^ to Fe^3+^. The fact that the peak only occurs in the second scan can be explained when iron in the hybrid material is present as Fe^3+^. The oxidation process becomes visible only after recycling to Fe^2+^. Therefore, the observed reduction is not only due to the reduction of oxygen, but there is also an underlying reduction of Fe^3+^. The CV indicates electrochemical activity and a quasi-reversible system.

Hybrid materials with iron oxides described so far are mainly iron oxides applied as layers onto carbon-based materials rather than both components present as a domain mixture [3,6,12,41]. Such layered hybrids have been successfully applied as anode materials for lithium-ion batteries [5,6,42,43,44] and in Fenton processes [45,46,47]. For the material with domain structures as well-mixed salt-and-pepper-type arrangements as produced in this study, the effect of graphite containment is expected to be more predominant with regard to performance.

## 4. Conclusions

In this study, a trend between process pressure and porosity of iron oxide nanomaterial was observed, allowing for control of microstructure during magnetron sputtering. Sputtered carbon could be produced as a defected graphite phase.

With alternated sputtering of iron oxide and graphite, a hybrid material with nanocontainment can be produced following typical Volmer–Weber growth. By incorporating sputtered graphite into the iron oxide nanomaterial, the disadvantages of the pure iron oxide material such as high resistance and instability can be circumvented. At the same time, the new hybrid material shows improved electrochemical activity as compared to pure iron oxide coatings. On further optimization, the new sputtering preparation method may be suited for the production of large-area roll-to-roll iron oxide–graphite materials for electrochemical applications such as water purification or energy storage.

## Data Availability

The data presented in this study are available on request from the corresponding author.

## Supplementary Materials

The following supporting information can be downloaded at: https://www.mdpi.com/article/10.3390/nano14030252/s1, Figure S1. XPS wide scan of iron oxide material deposited onto CF; Figure S2. Peak fit for Fe 2p peak; Figure S3. EDX mapping and colour SEM of the hybrid produced by magnetron sputtering, rec-orded at 10 kV with a magnification of ×65,000 (a) showing iron; (b) and carbon.
