# Supplementary material for: Magnetron Sputtering as a Versatile Tool for Precise Synthesis of Hybrid Iron Oxide–Graphite Nanomaterial for Electrochemical Applications
Source: Nanomaterials (Basel). 2024 Jan 24;14(3):252. doi: 10.3390/nano14030252 (PMC10856520; doi:10.3390/nano14030252)
Supplement: Supplementary file 1 [file nanomaterials-14-00252-s001.zip › nanomaterials-2751655-supplementary.pdf]

nanomaterials

Supplementary Materials

Magnetron Sputtering as a Versatile Tool for Precise Synthesis of Hybrid Iron Oxide-Graphite Nanomaterial for Electrochemical Applications

Fee Käufer [1,2,*], Antje Quade [2], Angela Kruth [2] and Heike Kahlert [1]

[1] University of Greifswald; hkahlert@uni-greifswald.de
[2] Leibniz Institute for Plasma Science and Technology; fee.kaeufer@inp-greifswald.de
* Correspondence: fee.kaeufer@inp-greifswald.de

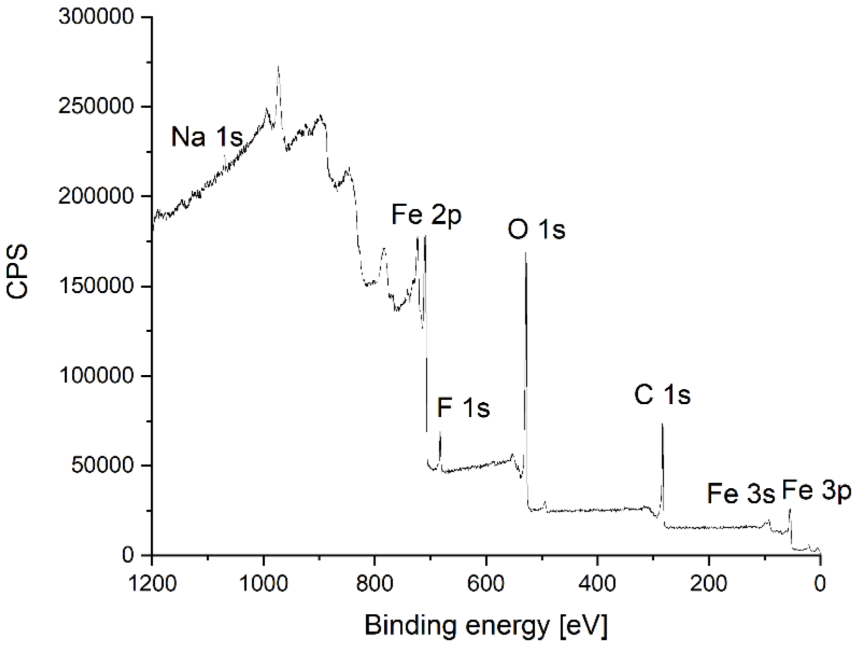

Figure S1. XPS wide scan of iron oxide material deposited onto CF.

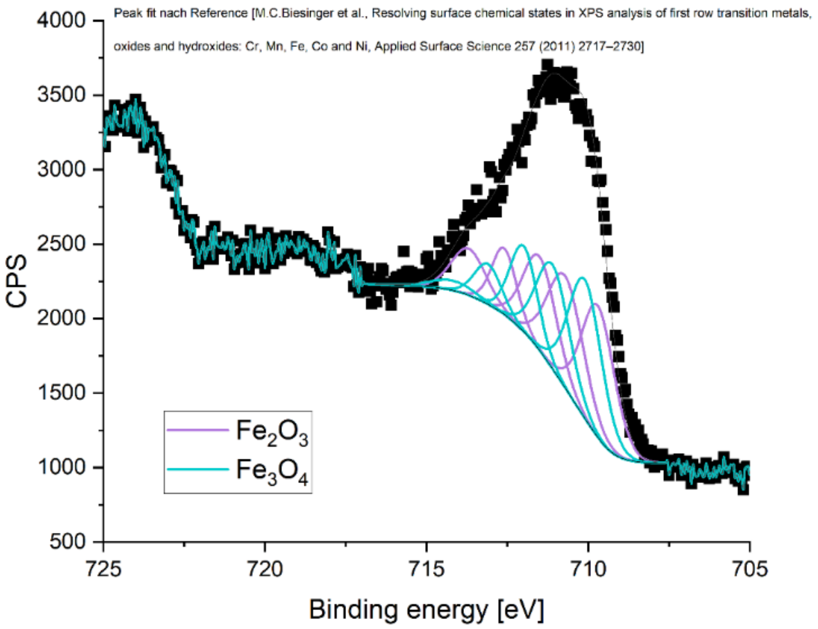

Figure S2. Peak fit for Fe 2p peak.

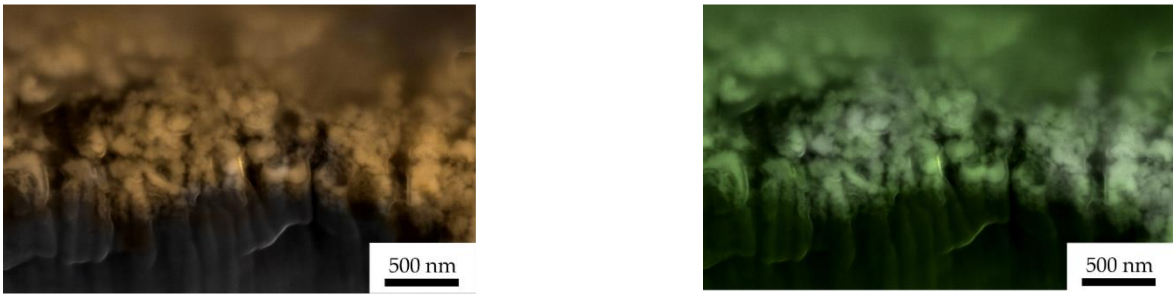

(**a**) (**b**)

Figure S3. EDX mapping and colour SEM of the hybrid produced by magnetron sputtering, recorded at 10 kV with a magnification of x65,000 (**a**) showing iron; (**b**) and carbon.
